# Genotypic and phenotypic analyses of two distinct sets of Pseudomonas aeruginosa urinary tract isolates

**DOI:** 10.1099/jmm.0.001971

**Published:** 2025-02-27

**Authors:** H. Ebrahim, S. Haldenby, M.P. Moore, A.A. Dashti, R.V. Floyd, J.L. Fothergill

**Affiliations:** 1 Department of Clinical Infection, Microbiology and Immunology, Institute of Infection, Veterinary and Ecological Sciences, University of Liverpool, Liverpool, UK; 2 School of Arts and Sciences, American International University, Kuwait; 3 Centre for Genomic Research, Institute of Integrative Biology, University of Liverpool, Liverpool, UK; 4 School of Life Sciences, University of Warwick, Coventry, UK; 5 Department of Medical Laboratory Sciences, Faculty of Allied Health Science, Health Sciences Centre, Kuwait University, Kuwait; 6 School of Life Sciences, University of Liverpool, Liverpool, UK

**Keywords:** antimicrobial resistance, biofilm formation, *Pseudomonas aeruginosa*, urinary tract infections, virulence factors

## Abstract

**Introduction.** Urinary tract infections (UTIs) are associated with a high burden of morbidity, mortality and cost. *Pseudomonas aeruginosa* employs a myriad of virulence factors, including biofilm formation and motility mechanisms, to cause infections including persistent UTIs. *P. aeruginosa* is highly resistant to antibiotics, and the World Health Organization has identified it as a pathogen for which novel antimicrobials are urgently required.

**Gap statement.** Genotypic and phenotypic characterization of *P. aeruginosa* from UTIs is underreported. In addition, the rise of antimicrobial resistance (AMR) is a cause for concern, particularly in many countries where surveillance is severely lacking.

**Aim.** To identify genotypic and phenotypic characteristics of *P. aeruginosa* UTI isolates sourced from the UK and the state of Kuwait, with an emphasis on genotypic diversity and AMR.

**Methods.** Twenty-three *P*. *aeruginosa* UTI isolates were sourced from the UK and Kuwait. To establish the phenotypes of UK isolates, growth analysis, biofilm formation assays, motility assays and antibiotic disc diffusion assays were performed. Whole-genome sequencing, antimicrobial susceptibility assays and *in silico* detection of AMR-associated genes were conducted on both sets of isolates.

**Results.** In terms of their phenotypic characteristics and genomic composition, the UTI isolates varied. Multiple resistance genes are associated with resistance to various classes of antibiotics, such as aminoglycosides, fluoroquinolones and *β*-lactams, particularly in isolates from Kuwait. Extreme antibiotic resistance was detected in the isolates obtained from Kuwait, indicating that the country may be an antibiotic resistance hotspot.

**Conclusion.** This study highlights that isolates from UTIs are diverse and can display extremely high resistance. Surveillance in countries such as Kuwait is currently limited, and this study suggests the need for greater surveillance.

## Introduction

Urinary tract infections (UTIs) are some of the most widespread infections in healthcare and community settings worldwide, with around 150 million patients infected annually [1,2]. According to the National Health Service in England, UTIs resulted in more than 800 000 hospital visits in the period between 2018 and 2023 [3]. UTIs are also one of the main causes of serious *Escherichia coli* (*E. coli*) bloodstream infections and a major contributor to the rise of antimicrobial resistance (AMR) in England [4].

*Pseudomonas aeruginosa* is a Gram-negative, motile, facultative anaerobic bacteria [5]. *P. aeruginosa* causes opportunistic infections in people with cystic fibrosis, cancer, burns, urinary tract complications and chronic wounds [6]. The World Health Organization (WHO) declared *P. aeruginosa* as a micro-organism of concern and in urgent need of new medical interventions [7]. Nosocomial infections caused by *P. aeruginosa* are often difficult to treat with antibiotics due to intrinsic and acquired multidrug resistance (MDR) [8]. *P. aeruginosa* also utilizes biofilm formation, which can complicate the treatment in respiratory, urinary tract and eye infections [9,10]. The bacterium utilizes a complex system of regulatory circuits via quorum sensing, two component systems and alternative sigma factors along with other regulators [11]. The large complex genome of *P. aeruginosa* (6–7 MB) contributes to adaptation in different environments and niches [12,13]. In addition*, P. aeruginosa* utilizes multiple virulence factors such as phenazines, elastase, phospholipase C, alkaline protease, rhamnolipids and hydrogen cyanide [14]. In a murine model of catheter-associated infections (CAUTIs), *P. aeruginosa* utilizes the type III secretion system to cause acute infections [15].

*P. aeruginosa* is efficient in causing acute or recurrent UTI, partially due to its ability to form biofilms and survive intracellularly [16]. The primary route to the urinary tract occurs in nosocomial settings, and UTIs account for 40% of hospital-acquired infections [5]. The percentage of CAUTIs attributed to *P. aeruginosa* is estimated at 35%, indicating that the pathogen is proficient at causing these infections [5]. The presence of patients in hospitals for more than 30 days increases the risk of acquiring UTIs by almost 100% [17]. Infections caused by CAUTI *P. aeruginosa* are usually known to be severe, persistent and antibiotic-resistant [18].

Whilst surveillance of the prevalence of AMR of UTI *P. aeruginosa* in the UK occurs, it is not comprehensive. Ironmonger *et al*. [19] conducted a comprehensive AMR surveillance in the Midlands region of the UK to identify resistance patterns of uropathogens, including *P. aeruginosa*. A total of 786 *Pseudomonas* spp. isolates (4.08% of all uropathogens) were acquired over a 4-year period from a population of 5.6 million, and 5.7% carbapenemase producers were identified [20]. However, many other phenotypic features are not reported. Furthermore, the similarity of these data to other countries worldwide is unclear. Minimal reporting on UTIs is common, including in the Gulf Cooperation Council (GCC), which is comprised of six countries: the State of Kuwait, the Kingdom of Saudi Arabia, the Kingdom of Bahrain, the State of Qatar, the United Arab Emirates and the Sultanate of Oman. Current evidence suggests that *P. aeruginosa* resistance to antibiotics is rapidly evolving in the GCC countries [21,22]. The increasing rates of AMR are attributed to several factors such as travel [23,24] and high use of antibiotics [25,26] including antibiotic sales from pharmacies without prescription [21,27]. Zowawi *et al*. conducted a regional study on *P. aeruginosa* clinical isolates and found that high-risk clones were widespread; in particular, *bla*_VIM-_ was detected in 39% of a total of 95 isolates [22]. Consistent surveillance on the spread of *P. aeruginosa* within Kuwaiti hospitals is lacking. Multiple studies reveal the existence of multidrug-resistant *P. aeruginosa* in nosocomial settings. In 1997, a study reported ten UTI *P. aeruginosa* isolates with aminoglycosides being the most commonly resisted class of antibiotics. A study conducted over a 3-year period (2005–2007) in one of the largest hospitals in Kuwait revealed that 9.8% of *P. aeruginosa* UTIs were acquired in the hospital, compared to 4.33% isolated from outpatients, and 15 and 14% of hospital-acquired isolates were resistant to amikacin and piperacillin/tazobactam, respectively [28]. However, these published reports lack detailed genomic analyses on the carriage of AMR genes.

In this study, we highlight the genotypic and phenotypic characteristics of *P. aeruginosa* UTI isolates obtained in the UK and compare them to a small subset of isolates from the State of Kuwait.

## Methods

### Bacterial isolates

*P. aeruginosa* isolates were obtained from the Royal Liverpool University Hospital in Liverpool (*n*=15) and the State of Kuwait (*n*=8). In the UK panel, the average age of patients upon bacterial isolation was 70 years old with 47% isolates from women (Table 1). All isolates sourced from Kuwait were isolated in 2005 with no further information available.

**Table 1.** *P. aeruginosa* isolates used in this study

Isolate name	Source	Date	Country of origin	Gender	Age	Reference
PAO1 (ATCC 15692)	Wound	1954	Australia	na	na	[73]
PA14 (NR-50573)	Burn wound	1977	USA	na	na	[74]
133042	Urine	11 October 2013	UK	M	52	This study
133043	Urine	16 October 2013	UK	F	80	This study
133044	Urine	16 October 2013	UK	F	69	This study
133065	Urine	18 October 2013	UK	F	70	This study
133075	Urine	24 October 2013	UK	M	76	This study
133082	Urine	25 October 2013	UK	M	89	This study
133083	Urine	25 October 2013	UK	F	68	This study
133090	Urine	25 October 2013	UK	M	83	This study
133098	Urine	31 October 2013	UK	M	58	This study
133099	Urine	31 October 2013	UK	F	61	This study
133104	Urine	01 November 2013	UK	M	72	This study
133105	Urine	01 November 2013	UK	M	50	This study
133106	Urine	02 November 2013	UK	M	83	This study
133117	Urine	02 November 2013	UK	F	61	This study
133126	Urine	07 November 2013	UK	F	75	This study
758	Urine	04 January 2005	Kuwait	na	na	This study
783	Urine	10 January 2005	Kuwait	na	na	This study
786	Urine	11 January 2005	Kuwait	na	na	This study
864	Urine	18 April 2005	Kuwait	na	na	This study
888	Urine	15 May 2005	Kuwait	na	na	This study
902	Urine	22 May 2005	Kuwait	na	na	This study
925	Urine	05 June 2005	Kuwait	na	na	This study
1083	Urine	12 November 2005	Kuwait	na	na	This study

### Isolate storage and culture

The isolates were stored at −80 °C in Luria–Bertani (LB) broth (Sigma-Aldrich) (w/v) with 10% glycerol (Sigma-Aldrich). Isolates were streaked onto LB agar (Appleton Woods) and grown overnight at 37 °C. A single colony of each *P. aeruginosa* isolate was utilized to inoculate LB broth (Sigma-Aldrich) in universal tubes overnight. Subsequently, the cultures were grown at 37 °C shaking at 180 r.p.m.

### Growth analysis

To establish the rate of bacterial growth, overnight cultures were diluted 1 : 100 in polystyrene 96-well plates (Corning® Costar®). The assay was performed with four technical replicates and in quadruplicate biological replicates. The growth of each well was monitored for 24 h at 37 °C, and the absorbance at 600 nm was recorded every 30 min by the FLUOstar Omega plate reader. Analysis was performed using Growthcurver in RStudio.

### Initial biofilm attachment assay

The overnight cultures of the UK-based clinical isolates were diluted 1 : 100 in LB. This was followed by the addition of 200 µl of each *P. aeruginosa*-containing LB solution in quadruplicate to a 96-well plate (Corning® Costar®) and grown at 37 °C for 24 h or 48 h. After the incubation period, the broth was removed, followed by the application of 200 µl of PBS to the wells twice to wash the biofilms. Removal of PBS was conducted followed by drying at 37 °C. Two hundred microlitres of 0.25% crystal violet (CV) w/v in dH_2_O (Sigma-Aldrich) were added into each well for 10 min. Stained biofilms were solubilized by adding 200 µl of 95% ethanol v/v (Sigma-Aldrich) for 10 min and transferred to a new 96-well plate (Corning® Costar®). The absorbance was measured at OD_600_ nm on the FLUOstar Omega plate reader to determine the biofilm biomass.

### Motility assays

#### Twitching

To measure the twitching motility of the isolates, a colony of *P. aeruginosa* was stabbed in the middle of an agar plate to the bottom. The colonies were then grown overnight at 37 °C. The agar was then removed, and the plate was subsequently stained with 0.25% CV for 15 min. Twitching zones were measured using a ruler. The cut-off point in which the isolate was considered motile was 5 mm in diameter.

#### Swimming motility

The methodology used is based on a swimming motility assay by Ha *et al.* [29]. Briefly, the media was prepared according to the protocol described. *P. aeruginosa* cultures were grown overnight in LB broth at 37 °C shaking at 180 r.p.m. Subsequently, a sterile toothpick was used to stab the agar from the top to near the bottom of the plate. The agar plates were incubated at 37 °C for 18 h. Three biological replicates were performed for each isolate in the study. Millimetre measurements were taken to assess the diameter of the swimming bacteria. To determine whether the isolate is motile, comparisons with the reference laboratory strain PA01 were conducted, and a cut-off of 6 mm was used to classify isolates as non-motile.

#### Swarming motility

The swarming motility test was performed according to the protocol established by Ha *et al.* [29]. Once the preparations of plate cultures were completed, 2.5 µl of overnight *P. aeruginosa* cultures was added to the top of the agar. The agar plates were incubated overnight at 37 °C for 18 h. Three biological replicates were conducted for each isolate. Measurements in millimetre were taken, and the isolates were deemed motile/non-motile depending on the diameter of 6 mm.

### Antibiotic resistance and genomic sequencing

#### Disc diffusion assay

Antibiotic susceptibility testing was performed based on the European Committee on Antimicrobial Susceptibility Testing (EUCAST) protocol [30]. Three biological replicates of each clinical isolate were grown overnight on Mueller–Hinton agar with an antibiotic disc (Thermo Fisher Scientific). The measurements in millimetre were determined and calculated as an average of three readings and compared to the published EUCAST breakpoints to determine if the clinical isolate is susceptible, has increased exposure or is resistant. The ‘I’ category is a second level of susceptibility, where there is a high likelihood of therapeutic success if the exposure to the agent is increased. Susceptibility to tobramycin, gentamicin, amikacin, netilmicin, imipenem, meropenem, doripenem, ceftazidime, cefepime, ceftazidime/avibactam, levofloxacin, ciprofloxacin, aztreonam, piperacillin, piperacillin/tazobactam, ticarcillin and ticarcillin/clavulanic acid was tested.

#### MICs of antimicrobial agents

MICs for colistin using one clinical UTI *P. aeruginosa* isolate 758 sourced from Kuwait were determined using the microdilution method as described in the guidelines of EUCAST [31]. Briefly, 100 µl of overnight culture of the isolate was diluted to OD_600_ of 0.05 and was mixed with 100 µl of serially diluted colistin (512–0.5 µg ml^−1^) (Sigma, UK) in triplicate using cation-adjusted Mueller Hinton broth. Microtitre plates were incubated for 1–2 days at 37 °C without shaking, and bacterial growth was determined by measuring absorbance at OD600 with a FLUOstar® Omega microplate reader and the MARS Data Analysis Software test.

#### Genomic DNA analysis

After extraction with the Wizard® Genomic DNA Purification Kit (Promega), all isolates in this study were sequenced. The DNA was preserved between 2 and 8 °C in preparation for sequencing at the University of Liverpool’s Centre for Genomic Research (CGR). Specifically, two isolates were sequenced for each panel sample. Using an Illumina HiSeq 2000 sequencer, 100 bp paired reads were produced from the ends of 500 bp fragments. Upon detection of Illumina adapter sequences using Cutdapt version 1.2.1 [32], FASTQ files containing sequenced read data were truncated. This was followed by the selection of option -O3, which resulted in the 3′ ends of all reads matching the adapter sequence by at least 3 bp being trimmed. The Sickle version 1.2 was utilized, and a minimum window quality of 20 was selected as the score for additional sequence trimming [33].

#### Identification of antibiotic resistance genes

The Comprehensive Antibiotic Resistance Database (CARD; http://arpcard.mcmaster.ca) was used to look for resistance genes on the CGR-obtained sequences [34]. Prior to analysis, the sequences were entered into the Resistance Gene Identifier (RGI) search tool, and the following options were checked: DNA sequence, perfect and strict hits only, exclude nudge and high quality/coverage.

#### Statistical analysis

All statistical analyses (unless otherwise specified) were conducted using Prism 9.5.1 and RStudio. The Shapiro–Wilk test was used to assess the distribution. All assays and growth curves were analysed with Kruskal–Wallis analysis of variance and Dunnett’s post-hoc test for nonparametric data.

## Results

### Growth dynamics of *P. aeruginosa* UTI isolates in nutrient-rich media

**Fig. 1.**
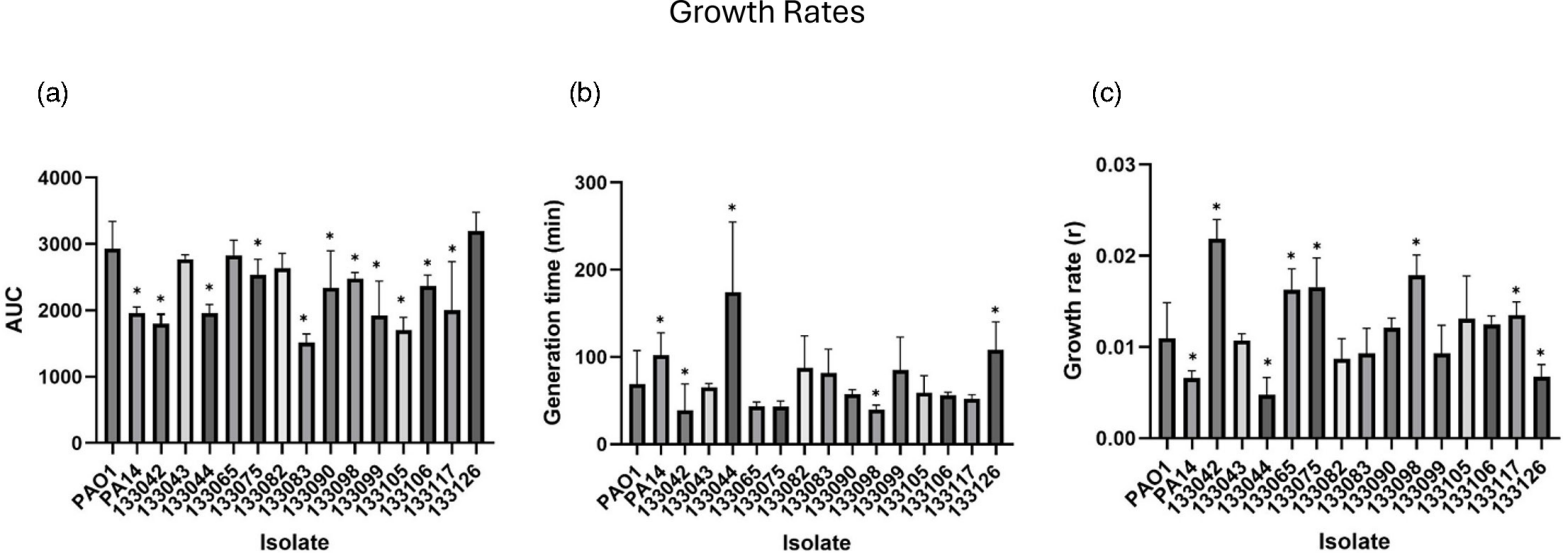
Growth of UTI isolates and two reference strains (PAO1 and PA14) over 24 h (*n*≥10). OD was measured at OD600 and analysed using Growthcurver to produce (a) AUC, (**b**) generation time and (c) growth rate. ANOVA and Dunnett’s multiple comparison tests were performed between each isolate compared to PAO1. Graphs show individual data points, mean and sd, analysed with a Kruskal–Wallis ANOVA and Dunnett’s multiple comparison tests. *P<0.05.

Growth in LB media was monitored for 24 h for the reference strains and the 14 UK-based UTI isolates. The area under the curve (AUC) was lower than PAO1 for all isolates, and for 10 out of 14 UTI isolates, this was significantly reduced (Fig. 1). However, the UTI isolates exhibited a high degree of variability in the generation time and overall growth rate. Two UTI isolates had a significantly slower generation time (133044 and 133126, *P*<0.0001 and *P*=0.009, respectively), and two displayed a significantly faster generation time (133042 and 133098, *P*=0.041 and *P*=0.039, respectively). Five UTI isolates had a significantly higher growth rate, whilst two isolates were significantly reduced (Fig. 1). This highlights the inter-isolate variation. However, as PAO1 is considered well adapted to the laboratory environment, it was notable that many of the UTIs displayed similar or faster growth under these conditions.

### Biofilm formation

**Fig. 2.**
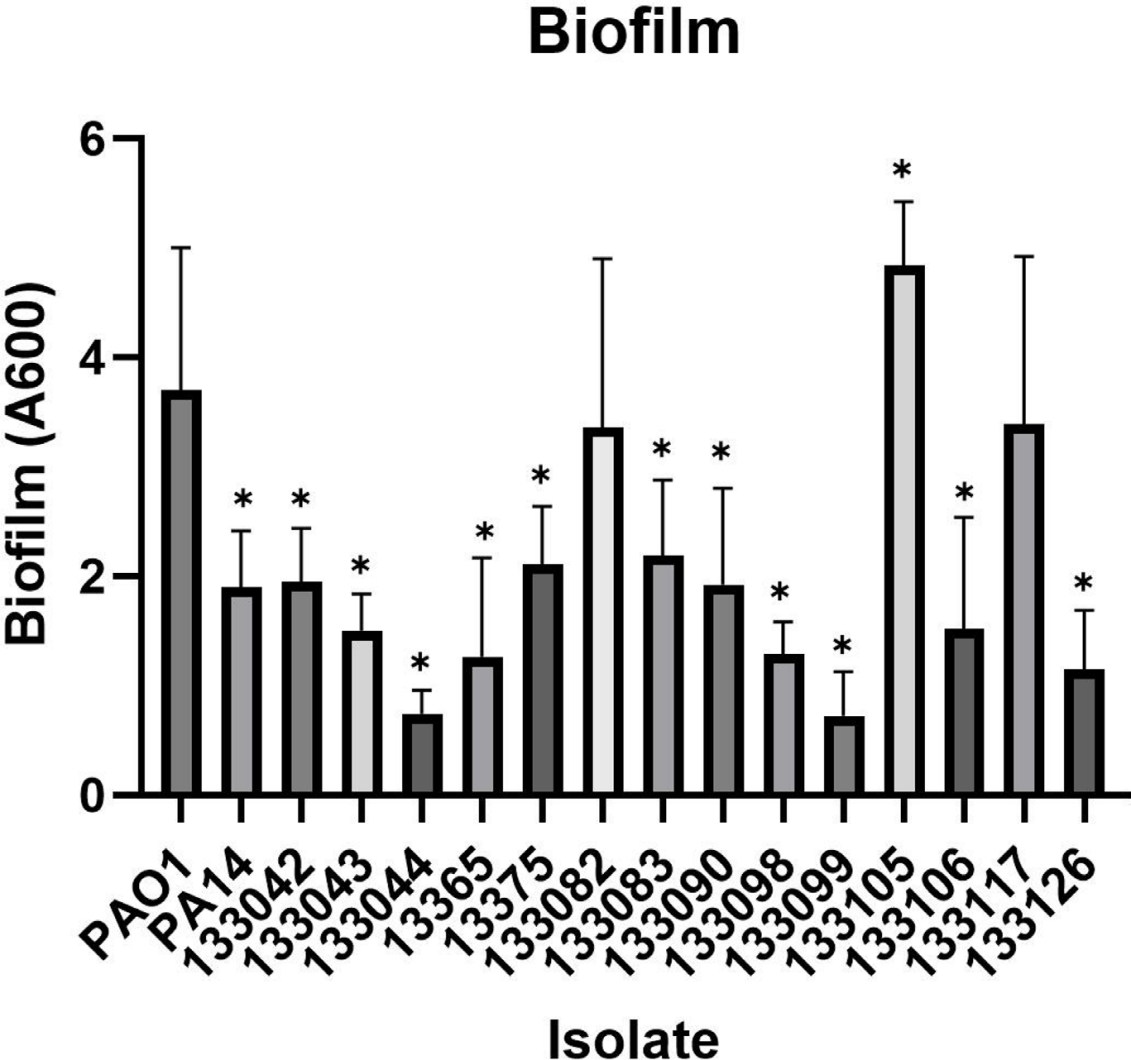
Biofilm formation in the UK-based isolates and reference strains. ANOVA and Dunnett’s multiple comparison tests were performed between each isolate compared to PAO1. Individual data points, mean and sd analysed with a Kruskal–Wallis ANOVA and Dunnett’s multiple comparison tests are shown. *P<0.05.

In order to compare biofilm capability, initial attachment on polystyrene wells was quantified using CV staining. Twelve of the 14 UTI isolates displayed a significantly lower level of biofilm compared to the reference strain PAO1, with isolate 13044 showing the lowest biomass (*P*<0.0001) (Fig. 2). The results show that though all isolates showed evidence of biofilm formation, many were significantly reduced compared to PAO1.

### Bacterial motility of UTI isolates

#### Swimming motility

**Fig. 3.**
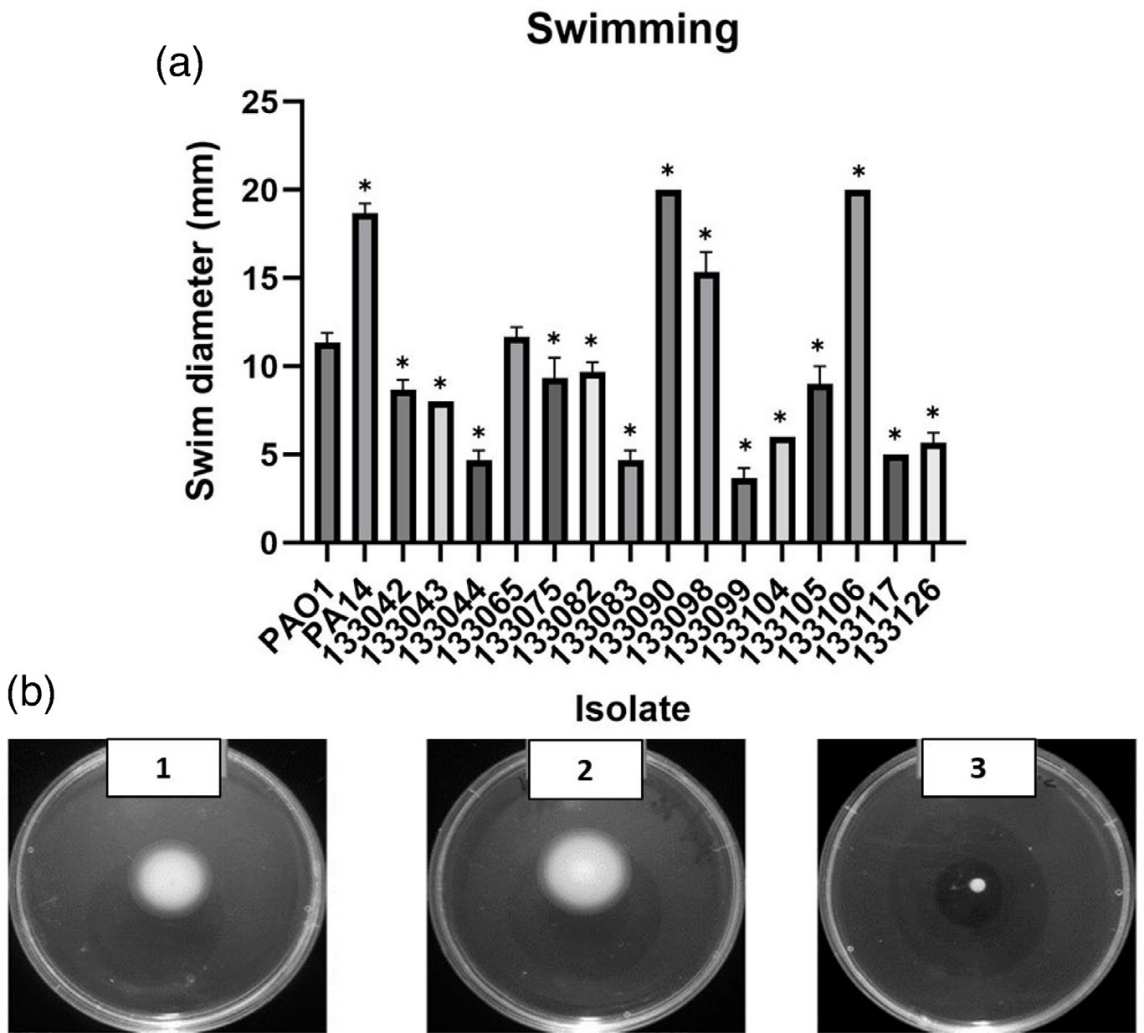
Swimming of *P. aeruginosa* UTI isolates. (**a**) Distances (mm) travelled by each isolate. ANOVA and Dunnett’s multiple comparison tests were performed between each isolate compared to PAO1. Individual data points, mean and sd analysed with a Kruskal–Wallis ANOVA and Dunnett’s multiple comparison tests are shown, compared to PAO1. *P<0.05. (**b**) The images depict the swimming motility of PA14 (image 1), isolate 133106 (image 2) and isolate 133099 (**image 3**).

Swimming motility is conducted by a rotating polar flagellum, and flagella are also considered virulence factors, which promote biofilm formation [35]. A plate-based method was utilized to assess the swimming ability of the 15 UK-based isolates [29]. Upon comparison with the reference strain PAO1 and using a cut-off value of 5 mm, 80% of the isolates showed the ability to swim through an aqueous medium (Fig. 3). Isolates 133044, 133083 and 13099 were classed as non-motile; they were beneath the cut-off value of 6 mm and showed a significant reduction in motility compared to PA01 (all *P*<0.0001).

#### Twitching motility

**Fig. 4.**
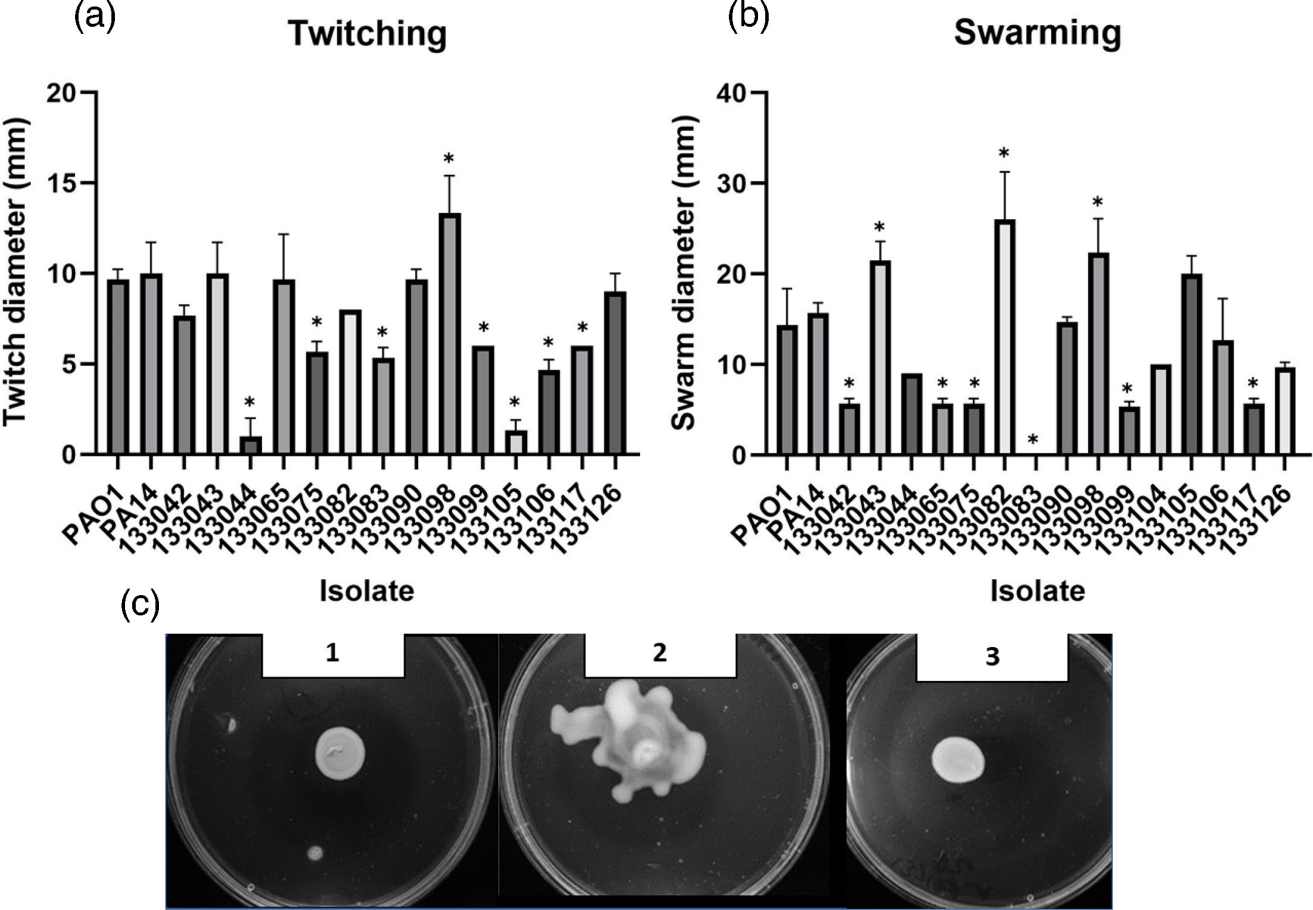
(a) Twitching motility. The cut-off for twitching was 5 mm cut-off motility. (**b**) Swarming motility, the mean average of distance travelled (mm) by swarming motility. The cut-off value was 6 mm. ANOVA and Dunnett’s multiple comparison tests were performed between each isolate compared to PAO1. Individual data points, mean and sd analysed with a Kruskal–Wallis ANOVA and Dunnett’s multiple comparison tests are shown. *P<0.05. (**c**) Plate images of swarming motility. Image 1: PAO1. Image 2) : isolate 133098. Image 3: isolate 133082 with moderate swarming ability.

In contrast to swimming motility, twitching motility is conducted by type IV pili. The pili facilitate the movement of *P. aeruginosa* across surfaces and aid colonization of the host and biofilm formation [36]. The mean distance travelled by all the 15 clinical isolates was 6 mm sd=+/−0.75 mm (Fig. 4a). The cut-off (5 mm) was exceeded by all the UTI isolates except 133044 and 133105, which were non-motile (1 mm). Therefore, the percentage of the isolates capable of performing twitching motility is 86.6%.

#### Swarming motility

Swarming motility is utilized by *P. aeruginosa* to achieve fast movement across semi-fluid surfaces [37]. The cut-off point used to define the motile and the non-motile *P. aeruginosa* was 6 mm (Fig. 4b, c). The mean distance travelled by all isolates was 6 mm. With the exception of two isolates (133083 and 133099), all UK-based UTI isolates were capable of swarming. Three isolates (133043, 133082 and 133098; *P*=0.0222, *P*<0.0001 and *P*=0.0025, respectively) were significantly increased in swarming compared to PAO1.

#### Antibiotic susceptibility in clinical UTI isolates

In order to test the susceptibility of *P. aeruginosa* to clinical therapeutics, an extensive panel of antibiotics was utilized to examine all clinical isolates and reference isolates PAO1 and PA14 according to clinical breaking points set by EUCAST [38]. Table 2 displays the susceptibility testing of the UK isolates. Overall, one isolate (133044) displayed multidrug resistance (defined by resistance to one antibiotic in three or more classes). All UK isolates were susceptible with increased exposure to penicillins. For cephalosporins, all isolates were susceptible with increased exposure to ceftazidime and cefepime, and fully susceptible to ceftazidime/avibactam. One isolate (133044) was resistant to tobramycin, with evidence of resistance to other aminoglycosides, but no breakpoints are defined for these. No zones of inhibition were observed when 133044 was exposed to tobramycin, netilmicin or gentamicin. Carbapenems are a key, last-resort antibiotic for many *P. aeruginosa* infections. One UK UTI clinical isolate (133044) displayed resistance to imipenem; however, the majority of isolates were fully susceptible to meropenem. One isolate (133105) was resistant to aztreonam, with no zone of inhibition (Table 2).

**Table 2.** Antimicrobial susceptibility of *P. aeruginosa* isolates from UTIs in the UK and Kuwait. The size in millimetre refers to the zone of inhibition following disc diffusion assays. Resistance (R) is shown in red and susceptibility in white, and isolates displaying the intermediate resistance group termed increased exposure (I) are shown in yellow

The highest prevalence of resistance was to ciprofloxacin, a fluoroquinolone antibiotic utilized to treat *P. aeruginosa* infections. Of the 14 isolates, ten were resistant to this antibiotic with the remainder deemed susceptible with increased exposure. For four isolates, there was no zone of inhibition to ciprofloxacin, and three of these also showed complete resistance to another fluoroquinolone, levofloxacin.

Resistance in the UK isolates was compared to a limited collection of resistant isolates from Kuwait. In contrast to the UK isolates, the isolates from Kuwait displayed very high levels of resistance (Table 2), albeit there appears to be bias in the selection of the panel. Seven out of eight isolates studied displayed multidrug resistance. Two isolates (758 and 783) were resistant to all antibiotics for which breakpoints were available. A broth microdilution MIC was performed to determine whether isolates 758 and 783 were resistant to the polymyxin colistin. Colistin was effective to both isolates at 2 mg l^−1^, indicating that it might be utilized as a last-resort antibiotic to treat these strains. These isolates must be regarded as having a significant potential for pan resistance, since the obtained value of 2 mg l^−1^ indicates the threshold for classification as sensitive (sensitive <2 and resistant >2).

Isolate 786 was only susceptible (increased exposure) to imipenem and meropenem. The other isolates displayed resistance to many classes on antibiotics. Isolate 1083 was the least resistant with resistance only to cefepime and ciprofloxacin.

#### Genomic DNA analysis

**Fig. 5.**
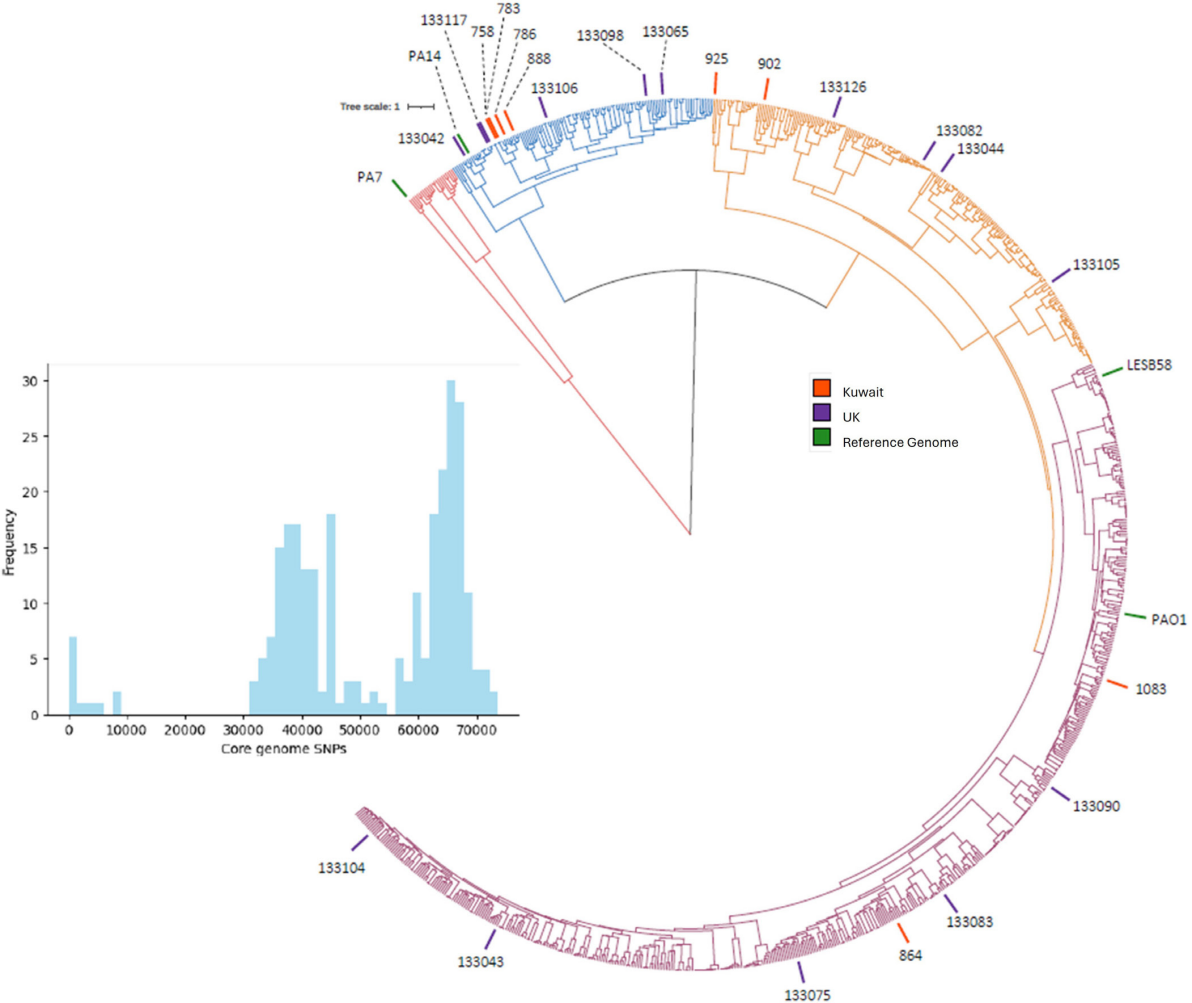
Phylogenetic tree of isolates from the UK and Kuwait based on their genomic composition, based on a core SNP phylogenetic tree using sequenced *P. aeruginosa* isolates from principal strains [40], is colour-coded in green; UK isolates are shown in purple, and Kuwaiti isolates are shown in red. The histogram shows the core genome SNP distances for the UTI isolates only (https://doi.org/10.6084/m9.figshare.27280527.v1).

To investigate the genomic context of UTI isolates in this investigation, whole-genome sequencing was used for the core SNP phylogeny of isolates from the UK and Kuwait. Unusually, group 2 (PA14-like) isolates were more prevalent in the clinical UTI isolates. This contrasts with the prevalence of group 1 (PAO1-like) isolates within the Pseudomonas.com database [39]. However, due to the small size of the panel, the results may not be representative of the wider *P. aeruginosa* clinical UTI population. To demonstrate the distribution of these UTI isolates amongst the wider population, a phylogenetic tree was created using sequenced isolates from Kos *et al*. (Fig. 5) [40]. Three Kuwaiti isolates clustered on the same branch of the phylogenetic tree, indicating that they are closely related.

#### Carriage of resistance genes in UTI isolates

CARD was used to identify resistance-associated genes in the UK UTI isolates. AAC [3]-IV was present only in isolate 133044. The gene encodes an aminoglycoside 3-*N*-acetyltransferase enzyme, which inactivates aminoglycosides including gentamicin and tobramycin, through enzymatic acetylation. Isolate 13044 displayed resistance to aminoglycosides. Further genes associated with aminoglycoside resistance were also identified including APH(3')-Ib, APH [4]-Ia (both plasmid-encoded aminoglycoside phosphotransferase in *E. coli*), APH [6]-Id (a mobile genetic element-encoded aminoglycoside gene) [41] and APH(3’)-IIb, and a chromosomal-encoded aminoglycoside [42] was also identified in this isolate.

Three isolates carried the gene *crpP* [43], which was linked to ciprofloxacin resistance; however, this has been questioned, and only one out of three of these isolates was resistant to ciprofloxacin [44]. In contrast, a *gyrA* mutation was identified in two isolates (133117 and 133099), and this corresponded to high-level resistance to both ciprofloxacin and levofloxacin. dfrB1 was identified in one isolate (133065) and encodes a plasmid-associated trimethoprim-resistant dihydrofolate reductase. A *Psuedomonas*-derived cephalosporinase (PDC) variant of extended-spectrum beta-lactamase was identified in all isolates [45].

The isolates from Kuwait were highly resistant. AAC(6’) family genes of aminoglycoside 6′-*N*-acetyltransferase enzymes, which inactivate gentamicin and tobramycin through enzymatic acetylation, were identified in 50% of the isolates with one isolate (786) carrying AAC(6’)-Ib7, a plasmid-encoded aminoglycoside acetyltransferase in *Enterobacter cloacae* and *Citrobacter freundii*. The isolates from Kuwait were highly resistant, with extensively drug-resistant isolates 758 and 783 being the most resistant in both collections (Table 3). These isolates contained the genes for the aminoglycoside transferase enzyme AAC (6')-ii and the aminoglycoside adenylyltransferase aaA61, which both enzymatically inactivate aminoglycoside antibiotics. In addition, both isolates contained the plasmid-encoded *E. coli* phosphotransferase APH(3')-Ib. This gene was detected in additional Kuwaiti isolates (786, 888 and 925) with similar aminoglycoside resistance. Isolate 888 contained five aminoglycoside resistance genes: AAC (6')-ii, aadA11, ANT(2’)-Ia, APH(3')-Ib and APH(3')-Via. Only isolate 133044 in the UK panel contained the aminoglycoside resistance genes APH(3’), APH [4], APH(3')-Iib and APH [6]-Id. This result demonstrates a correlation between genotype and susceptibility phenotype. Isolates 133042, 133075, 133117 and 925 all contained quinolone resistance genes, *crpP*. Chávez-Jacobo *et al*. [43] described the enzyme product CrpP as a novel ciprofloxacin-modifying agent. Only one isolate, 133117 (*crpP*+), exhibited ciprofloxacin resistance in disc diffusion testing. Isolates from Kuwait had the highest frequency (62.5%) of mutations in *gyrA* that cause fluoroquinolone resistance.

**Table 3.** AMR genes identified in *P. aeruginosa* UTI isolates through whole-genome sequencing and analysis using CARD. Percentage sequence identity is reported. These genes are involved in promoting resistance to multiple classes of antibiotics such as aminoglycosides (blue), fluoroquinolones (green), trimethoprim (yellow) and *β*-lactamases (orange) in the UK and Kuwait isolates with resistance induced by chromosome (C), integrons (I), transposons (T) and integrative element (IN)

Genes encoding *β*-lactamases are detected in all the UK and Kuwaiti isolates. OXA-50 is encoded chromosomally and is present on all isolates. The other identified OXA type was OXA-10, a resistance gene detected in only one isolate (786). *P. aeruginosa* contains class C AMR genes encoding PDC lactamases [45]. All of the isolates contained at least one PDC enzyme, although the types varied. PDC-1, PDC-2, PDC-3, PDC-4, PDC-5, PDC-7, PDC-8, PDC-9, PDV-10 and PDC-86 were detected. PDC-2 is present in 26% of all tested UTI isolates, followed by PDC-3, which was only detected in isolates 758, 783, 778 and 888 from Kuwait. PDC-5 and PDC-9 were unique to the UK isolates, with the former detected in 133044, 133082 and 130083 and the latter in 133042, 133099 and 133117. The remaining isolates contained PDC-1 (133090 and 864) as well as PDC-10 (133065 and 133098), PDC-7 (133106) and PDC-86 (902).

A single Verona integron-encoded metallo-*β*-lactamase (VIM) type gene was identified in three isolates. The VIM-28 gene was detected in these *P. aeruginosa* isolates (758, 783 and 1083).

## Discussion

Understanding UTIs is of paramount importance, as UTIs are one of the most common bacterial infections worldwide. These infections can cause severe discomfort and pain to those afflicted, leading to a reduced quality of life, and if left untreated, UTIs can progress to serious conditions, such as urosepsis [46]. Additionally, the overuse and misuse of antibiotics in UTI treatment contribute to antibiotic resistance, which is a global health crisis. *P. aeruginosa* is largely understudied in the UTI context but can result in complex and resistant infections. By gaining an insight into phenotypic and genotypic variation, effective treatment strategies, preventive measures and insights into the broader issue of antibiotic resistance may be gained.

*P. aeruginosa* can cause both acute and chronic infections. Chronicity is often associated with decreased growth under laboratory conditions. Here, the behaviour and fitness of UTI isolates were established by studying their growth under laboratory conditions. The majority of UTI isolates (71%) displayed a decreased AUC. Despite this, the generation time was relatively rapid with the majority of isolates showing faster generation than PAO1. Two isolates had a much slower generation time. This may reflect long-term adaptation to the urinary environment. However, no information is available regarding the length of infection, and therefore, this cannot be confirmed. Overall, the capacity of UTI isolates to thrive in the laboratory environment is heterogeneous; although, many isolates display fast growth dynamics akin to environmental or acute clinical isolates.

*P. aeruginosa* displays an excellent capacity to form biofilms on both abiotic and biotic surfaces. All the UTI isolates showed some degree of biofilm ability; however, 79% displayed significantly decreased biofilm compared to PAO1. Multiple studies have documented the ability of *P. aeruginosa* isolated from multiple infection sites to form biofilms [47,48]. *P. aeruginosa* can cause middle ear infections in cholesteatoma patients [49,50]. Eighty-three percent of otitis media *P. aeruginosa* isolates are efficient biofilm producers, with substantially higher levels of biomass produced than PAO1. The capacity to form biofilms may contribute to persistent infections in cholesteatoma patients [51]. Similar to the UTI isolates in this study, PAHM4, an isolate from a person with non-cystic fibrosis (CF) bronchiectasis, produced substantially less biofilm than PAO1 [52]. A study of 101 keratitis isolates revealed that isolates produced between 17 and 242% of the PAO1 control and that the ability to form biofilms was associated with poorer clinical outcomes [53]. Schaber *et al*. [54] compared five quorum sensing(QS) deficient isolates from respiratory, cutaneous and UTI infections to PAO1 to evaluate biofilm formation. The UTI isolate CI-5 was obtained from an 82-year-old patient who developed sepsis due to CAUTI. CV staining and comparison of all isolates revealed that, among all QS deficient isolates, the UTI isolate produced the highest biomass and up to 82% capacity of PAO1 [54]. In this study, two isolates (133117 and 925) had loss of function mutations in the key QS gene, *lasR*. A study comparing the capacity of uropathogenic *P. aeruginosa* serotypes to form biofilms *in vitro* using CV staining revealed variation between various serotypes. In particular, O11 serotypes produced the greatest biofilms and were more likely to be antibiotic-resistant; in contrast, O6 strains produced the weakest biofilms [55]. Vipin *et al*. [56] evaluated the ability of CAUTI *P. aeruginosa* to produce biofilms; all isolates formed biofilms between 0.20 and 1.11 (OD580), indicating the diversity of these clinical isolates [56]. Additionally, Tielen *et al*. [57] conducted CV staining on 12 mid-stream urine isolates and 18 CAUTI isolates and found the latter more proficient at producing biofilms. The majority of isolates were found to be intermediate biofilm producers [57]. Our results suggest that mid-stream UTIs are not as proficient in forming biofilms as keratitis, otitis media and CAUTI isolates. The ability to form biofilms is heterogeneous in the tested panel.

*P. aeruginosa* employs motility as a prominent virulence factor to aid infection in multiple niches [58]. Tielen *et al*. [57] reported that 100% of 12 UTI isolates were capable of swimming, in comparison to 16 out of 18 CAUTIs (88.8%). Loss of swimming function might be related to evolutionary adaptation mechanisms in chronic infections similar to those observed in the lungs of CF patients [57]. More than 95% of CAUTI isolates have the functional ability to swim according to Ruzicka *et al*. [59]. In this study, 80% of the UTI isolates exhibited the ability to swim. Swimming motility can allow bacteria to colonize new niches and initiate attachment and adherence on surfaces, contributing to biofilm formation including on catheter surfaces [60]. Type IV pili facilitate twitching motility, which is used to traverse solid and semisolid surfaces [61]. Seventy-five percent of the 139 *P*. *aeruginosa* UTI isolates examined by Ruzicka *et al*. [59] for twitching motility were found to be capable of twitching [59]. Winstanley *et al*. [62] examined the twitching motility of 63 keratitis isolates; 90% of the isolates demonstrated the capacity to twitch *in vitro* [62]. In addition, mutants lacking twitching motility were incapable of colonizing the cornea [63]. In our study, 86.6% of the isolates were able to twitch, indicating that this is a common characteristic of UTIs. Lack of twitching motility was associated with mutations in *pil* genes. Furthermore, 86% of UTI isolates tested in this study exhibited a high level of swarming motility. Hyper-motile strains that swarm tend to create flat biofilms [64]. There have been reports of up to 95% of *P. aeruginosa* CAUTI isolates with swarming motility [59]. Interestingly, swarming is a key characteristic of *Proteus mirabilis*, another urinary pathogen [65]. This may indicate that swarming is advantageous in the urinary environment.

The WHO has identified carbapenem-resistant *P. aeruginosa* as a critical issue requiring intervention [36]. Specific information on AMR of *P. aeruginosa* UTI isolates in both the UK and Kuwait is limited [19,28, 66]. Phenotypic resistance screening revealed a relatively low level of resistance in isolates from the UK. However, pan resistance was identified in some isolates from Kuwait. This could suggest that Kuwait may be a hotspot for antibiotic resistance; however, this small number of isolates was stored based on higher resistance and therefore represents a biassed subset. A recent study in a major Kuwaiti hospital revealed that 32.1% of *P. aeruginosa* isolates were MDR; the majority of these were derived from urine culture [67]. Extensive prospective studies should be conducted since most of the obtained AMR data in Kuwait are based on retrospective studies. Lack of surveillance programmes, inadequate use of antibiotics, travel and climate change all contribute to the spread of AMR in this region [21]. Whole-genome sequencing revealed resistance determinants in isolates from both geographical regions; however, uncommon resistance genes were identified in the genomes of isolates from Kuwait. The VIM family are able to inactivate beta-lactam antibiotics, and the unusual VIM-28 was identified in four isolates. VIM-28 is a metallo-*β*-lactamase initially reported in Egypt, and the structure of the VIM-28 gene contains an uncommon integron configuration, with the gene cassette located downstream of the ‘*intI1*’ gene. Egyptian nationals constitute one of Kuwait’s main ethnic minorities [68,69]. Travelling between the two nations may help explain the presence of ^bla-^VIM-28 in both regions. In addition to the intrinsic OXA-50 class D *β*-lactamase, which is present in all isolates, the OXA-10 class D *β*-lactamase was also detected in isolates 786 and 1083. To the best of our knowledge, this is the first report of OXA-10 in Kuwaiti bacterial isolates.

Multiple isolates exhibited resistance to aminoglycosides. Uncommon genes in *P. aeruginosa* including aadA6, aadA11 and aadA13 were observed. AadA-type genes encode aminoglycoside nucleotidyltransferases, and these are often encoded on mobile genetic elements such as plasmids and integrons. Genes for further aminoglycoside-modifying enzymes were also observed including AAC (6’)-ii, ANT(2'')-Ia and APH(3')-Ib. Fluoroquinolone resistance was also observed. Mutations in *gyrA* and plasmid-associated CrpP enzyme would contribute to ciprofloxacin and levofloxacin resistance in these isolates [43]. dfrB1 is a plasmid-associated, trimethoprim-resistant dihydrofolate reductase that was initially identified in *Bordetella bronchiseptica* bacteria [70], suggesting that it may have been horizontally transmitted to *P. aeruginosa*. Due to the extended resistance and identified genes, it is possible that these isolates contain MDR plasmids. Recent research on Thai isolates revealed that *P. aeruginosa* carries MDR megaplasmids [71]. Additional analysis, particularly using long-read sequencing data, would reveal further information on plasmid carriage. The implications of these findings are alarming and suggest that extensive AMR surveillance and management programmes would be beneficial.

In this study, only two MDR isolates were detected in isolates originating from the UK, indicating that they were largely susceptible to antibiotics. In general, the prevalence of AMR *P. aeruginosa* uropathogens is regarded as low [20]. This could be attributed to stricter antibiotic stewardship policies and increased public awareness of antibiotic misuse [72]. According to Ironmonger *et al.*, a 4-year surveillance investigation detected only 45 non-susceptible *P. aeruginosa* uropathogens to carbapenems amongst 6985 isolates from the Midlands region of England [20].

The findings in this study reveal the genomic and phenotypic diversity of *P. aeruginosa* isolates from UTIs. The data indicate that UTIs can be caused by a wide variety of phenotypic and genotypic combinations. The success of a given infection is therefore likely a complex interplay between bacterial characteristics and host factors, such as hospitalization and long-term catheter use. Sequencing conducted as part of this study detected the presence of AMR genes that could promote resistance to every class of antibiotics. Uncommon resistance genes were highly prevalent in the isolates from Kuwait, and this correlated with extremely high phenotypic resistance. However, the basis on which these isolates were selected is unclear and likely biassed. Thus, additional study would need to determine the prevalence of such strains. In addition, a deeper understanding of how *P. aeruginosa* responds to the urinary environment would allow for a more in-depth examination of the bacterial pathogenesis and aid in the identification of novel therapeutic interventions.

## Abbreviations

AMR: antimicrobial resistance
CARD: comprehensive antibiotic resistance database
CAUTIs: catheter-associated infections
CF: cystic fibrosis
CGR: Centre for Genomic Research
CV: crystal violet
EUCAST: European Committee on Antimicrobial Susceptibility Testing
LB: Luria–Bertani
UTI: urinary tract infection
VIM: Verona integron-encoded metallo-*β*-lactamase
WHO: World Health Organization
